# Comparison of 532 nm Micropulse Green Laser versus Continuous-Wave 532 nm Green Laser in Chronic Central Serous Chorioretinopathy: Long-Term Follow-Up

**DOI:** 10.1155/2020/4604567

**Published:** 2020-01-31

**Authors:** Katarzyna Piasecka, Piotr Gozdek, Mariusz Maroszyński, Dominik Odrobina

**Affiliations:** ^1^Ophthalmology Clinic Boni Fratres Lodziensis, Łódź, Poland; ^2^Faculty of Health Sciences, Jan Kochanowski Memorial University of Kielce, Kielce, Poland

## Abstract

The aim of this study was to analyze the efficacy of micropulse laser treatment (MLT) compared with the continuous-wave laser (CL) in treating eyes with chronic central serous chorioretinopathy (CSC) in a 12-month follow-up study. *Methods*: A retrospective observational study included 51 eyes with chronic CSC; 35 eyes were treated with MLT (Group A), and 16 eyes were treated with CL (Group B). We analyzed the best corrected visual acuity (BCVA) and retinal microstructural changes in spectral optical coherence tomography before the treatment, one and twelve months after the laser procedure. *Results*: The final mean BCVA was 0.89 ± 0.13 in Group A and 0.71 ± 0.17 in Group B. Photoreceptor length decreased significantly in both groups and amounted 61.2 *μ*m in Group A and 42.9 *μ*m in Group B one year after the treatment. Complete absorption of subretinal fluid twelve months after the laser procedure was noted in 74.3% eyes in Group A and in 87.5% eyes in group B. Hyper-reflective subretinal deposits were observed in 10/35 eyes in Group A but in 15/16 eyes in Group B on the final follow-up visit. *Conclusion*. MLT-treated patients showed better functional and microstructural results than patients treated with CL.

## 1. Introduction

Central serous chorioretinopathy (CSC) is an idiopathic, typically self-limited disease, in which an accumulation of subretinal fluid is observed. The pathophysiology remains unclear; CSC might be assigned to choroidal vessels' hyperpermeability, impairment of choroidal vascular autoregulation induced by steroids, catecholamines, or sympathomimetic agents, and dysfunction of retinal pigment epithelium (RPE) barrier and pumping [1]. Central serous neuroretinal detachment resolves spontaneously in over 80 to 90% of cases [2]. Nevertheless, in ten to twenty percent of the cases, the leakage RPE lingers over several months, leading to structural retinal impairment [3, 4]. Therefore, to avoid permanent moderate visual loss, metamorphopsia, or scotoma, safe and effective treatment is required.

Conventional continuous-wave laser (CL) is still one of the standard therapies to local leakage sites in chronic CSC, and its limitations associated with retinal scaring are, however, well described. Micropulse laser treatment (MLT) is an alternative nondestructive procedure which may also be useful in patients who experience a diffuse leakage or when the leakage is localized within or very close to the foveal avascular zone (FAZ).

The aim of this study was to analyze and compare the long term functional and anatomical results of 532 nm green laser MLT and CL treatment for chronic CSC.

## 2. Materials and Methods

Our study adhered to the tenets of the Declaration of Helsinki. Fifty-one patients with symptomatic chronic CSC (duration > 6 months) were included into this retrospective observational study. Each patient underwent complete ophthalmologic examination including best corrected visual acuity (BCVA) using standard Snellen eye charts, slit-lamp examination, and indirect ophthalmoscopy with Volk 78D. The presence of subretinal fluid was determined in fluorescein angiography (Spectralis; Heidelberg Engineering, Heidelberg, Germany) and confirmed by SOCT.

All eyes were examined by SOCT (Spectralis; Heidelberg Engineering, Heidelberg, Germany) with 3.9 *μ*m axial resolution and transverse resolution of 14 *μ*m. We performed horizontal line scan through the fovea and made 19 B-scans on an area of 4.5 × 6 mm in each patient. Three experienced examiners analyzed all B-scans and manually performed the measurements. We derived a medium value from three measurements in each case.

In SOCT, the fovea is recognized as the characteristic foveal depression where there is a lack of the internal retinal layers: nerve fiber layer, ganglion cell layer, inner nuclear layer, and inner plexiform layer. All measurements were performed in the central scan, where the fovea was recognized. We measured photoreceptor (PR) length defined as the distance between the external limiting membrane (ELM) and the most protruding outer segment of photoreceptors. Retinal thickness (RT) was defined as the distance between internal limiting membrane (ILM) and the most protruding outer segment of photoreceptors. We also recorded the value of subretinal fluid in the centre of the fovea and the presence of subretinal hiper-reflective deposits in the central scan.

BCVA and SOCT parameters were taken prior to treatment, one and twelve months after laser therapy.

Exclusion criteria were any invasive treatment due to CSC prior to the study, such as laser photocoagulation, photodynamic therapy, or anty-VEGF injections. We also excluded subjects with other ocular diseases such as diabetic retinopathy, retinal detachment, or any ocular inflammation.

Having obtained written informed consent from each patient, the laser treatment was performed by one ophthalmologist (DO). The leakage spots were determined in the earliest stage of fluorescein angiography.

MLT photocoagulation was carried out with a 532 nm micropulse green laser (IRIS IQ Medical, Iridex Corp., Mountain View, CA, USA) with a 200 *μ*m spot size, a 0.15 sec duration with 5% duty cycle (D/C), and 700–900 mW power. The accurate laser power for the MLT treatment was determined by the minimally visible laser scar of a trial photocoagulation created with continuous-wave laser energy for 0.15 s with a diameter of 200 *μ*m in the superotemporal quadrant, and 5% duty cycle of 400% of the threshold power was used. Three to four laser spots were applied to the leakage site defined in fluorescein angiography.

CL treatment was performed with a 532 nm green light laser (IRIS GL Medical, Iridex Corp., Mountain View, CA, USA). The spot diameter was 100–200 *μ*m, time was 0.10 sec duration, and the mean power was 150 mW (90–180 mW). Test spots in the superotemporal quadrant were made to obtain minimal retinal discoloration in order to determine the adequate laser power in each case.

We used Stata 12.1 Special Edition (StataCorp LP, College Station, Texas USA) for statistical analysis. Wilcoxon rank-sum test, Pearson's product-moment correlation coefficients, mixed-effects generalized linear models, and Fisher's exact test were used, and a *P* value of <0.05 was considered statistically significant.

## 3. Results

Thirty-five eyes of 35 patients with chronic CSC (mean age 48.5 ± 8.8 years) were treated with MLT and were included in Group A. CL treatment was performed on 16 eyes of 16 patients (mean age 48.8 ± 8.0 years), and they were enrolled in Group B.

Descriptive statistics for selected ophthalmological parameters in the studied subjects are shown in Table 1.

The patients' initial BCVA ranged from 6/19 to 6/7 (in decimals 0.3 to 0.9) in Group A and from 6/30 to 6/7.5 (in decimals 0.2 to 0.8) in Group B and did not differ significantly (mean and SD, respectively: 0.53 ± 0.16; 0.52 ± 0.20; *P* > 0.05, the Wilcoxon rank-sum test). The baseline BCVA did not statistically significantly correlate with the RT and PR length in either of the two study groups. Pearson's product-moment correlation coefficients for the BCVA at baseline for RT amounted *r* = –0.22 (*P*=0.207) in the MLT group, in the CL group *r* = 0.24 (*P*=0.376), and for PR: *r* = –0.06 (*P*=0.735) in Group A, *r* = –0.20 (*P*=0.455) in Group B.

BCVA improved significantly in both groups. At the final follow-up visit, mean BCVA was 0.89 ± 0.13 and 0.71 ± 0.17, respectively (*P* < 0.001). The increase in visual acuity was more pronounced in the MLT group compared with the second group (*P*=0.026, mixed-effects GLM).

The studied patients' age (in the MLT group only) negatively correlated with the amelioration of their visual acuity (*P*=0.027), that is, younger patients benefitted more from the MLT than their older counterparts.

Length of PR was similar in both groups prior to the treatment and decreased significantly after the laser procedure in all patients (Table 1). The decrease in the PR length in long term observation was less pronounced in the MLT group compared to the second group (61.2 *μ*m vs. 42.9 *μ*m; *P*=0.028, mixed-effects GLM). Taking the changes in the PR length and central RT into consideration, the patients' age did not condition the observed improvement in both traits (respectively, *P*=0.268 and *P*=0.668).

The pace of changes of subretinal fluid was similar in both study groups (*P*=0.474, mixed-effects GLM). Twelve months after the treatment, subretinal fluid amounted 12.4 *μ*m in MLT patients and 12.2 *μ*m in CL patients. During the first follow-up examination, one month after laser treatment, 19 out of 35 eyes (54.3%) in Group A showed complete absorption of subretinal fluid, whereas 5/16 (31.2%) eyes in Group B showed complete absorption of subretinal fluid (*P*=0.418, Fisher's exact test). Twelve months after laser treatment, 74.3% in Group A (26 out of 35 eyes) showed complete absorption of subretinal fluid, whereas 14/16 (87.5%) eyes in Group B showed residual fluid (*P*=0.870, Fisher's exact test).

**Figure 1:**
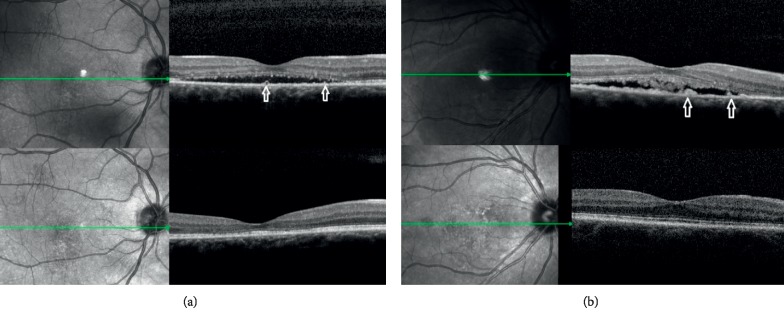
(a, b) Spectral optical coherence tomography images of eyes before treatment (top) and after MLT (bottom). Hyper-reflective subretinal deposits on retinal pigment epithelium layer are visible before but not after MLT (arrows).

Hyper-reflective subretinal deposits were demonstrated as a noticeable bulge in the RPE and were observed in 28/35 eyes in Group A and in 14/16 eyes in Group B before the treatment. On the final follow-up visit, RPE deposits were described in 10/35 eyes in Group A but in 15/16 eyes in Group B (Figure 1). The decrease in the number of deposits was significantly less pronounced in the CL group compared with the MLT group (*P* < 0.0001 Fisher's exact test). The depletion in deposits during the 12 months of observation was statistically significantly related to the shortening of photoreceptors in the MLT group only (*P*=0.006). In the CL group, the relationship was not statistically meaningful (*P*=0.121).

## 4. Discussion

To our knowledge, there are no published studies comparing the results of 532 nm green light subthreshold micropulse and continuous-wave laser treatment in CSC.

Untreated chronic CSC leads to various microstructural changes of the retina, such as the shortening of the outer nuclear layer, elongation of photoreceptor outer segments, RPE alterations, and foveal atrophy [3–5]. Conventional continuous-wave laser photocoagulation was recognized an effective treatment for both acute and chronic CSC in randomized controlled trials [6, 7]. Nevertheless, CL induces iatrogenic retinal damage and functional loss even when photocoagulation is accurately performed [8, 9]. As a result, the final visual and anatomical outcome is a result of degenerative changes derived from subfoveal fluid persisting more than 4 months prior to CL and retinal scaring after CL itself.

In a micropulsed diode laser, developed in 1990 by Pancratov, a train of very short laser pulses separated by variable quiet intervals is harnessed [10]. It allows for selectively targeting the RPE, sparing the photoreceptors and other intraretinal cells [10–12]. This nondestructive and thus noninflammatory procedure was successfully used in acute and chronic CSC [13, 14].

The results of diode subthreshold micropulse laser (810 nm) and micropulse yellow laser (577 nm) treatment have been reported in several studies proving that this therapy is safe and effective for CSC patients [15–19]. According to retrospective analysis of the PACORES group, yellow micropulse laser may be an adequate treatment alternative where PDT is not available [20].

Our retrospective observational study showed for the first time that green light MLT treatment is an effective treatment for CSC in one year of follow-up, providing a better functional and morphological outcome than a CL procedure.

In this study, BCVA was significantly improved at 1 month after treatment and remained stable at 12 months in both groups, yet the visual gain was significantly more pronounced in the MLT group. Our results are analogous with several short-time efficacy reports of other micropulse procedures with diode and yellow lights [13, 15]. Twelve months of observation allowed us to note further visual improvement in patients treated with MLT, which was not noted in the CL group. This compares favourably with the literature. At the 12 months of follow-up of 92 patients after 577 nm MLT, the mean BCVA increased from 0.4 to 0.63 when presented in Snellen charts [20]. But, in another study of long-term effects (17.82 ± 0.42 months of observation), Arsan reported further visual improvement after 577 nm subthreshold laser photocoagulation: the visual gain was from 0.44 at baseline to 0.87 ± 0.2 at the final visit [16].

We believe that the fact of delayed functional improvement is interrelated with microstructural retinal changes visualized in the SOCT examination. Analyzing the subretinal fluid absorption, final results showed no differences between the studied groups, although the effect was visible earlier in MLT-treated eyes. The ratio of complete resolution of subretinal fluid in the MLT group was 54.3% one month after treatment; thus, the results are similar to those reported in several small samples [15, 17–19]. The comparison of CL and MLT was presented in Maruko's study with yellow laser. In his patients, a complete resolution of SRF was seen in 66.7% after CL and in 64.3% after MLT procedures [15]. At the final visit in our study, 74.3% of MLT-treated eyes showed complete absorption of subretinal fluid. It is less than that in long-time observation of 17 months of Arsan, where he reported over 90% patients with complete fluid resolution [16].

Another interesting finding is that the length of photoreceptors varied between the MLT and CL groups in time. As it was previously reported by Matsumoto et al., photoreceptors elongate in chronic CSC, which may be an effect of the lack of normal photoreceptor phagocytosis by the RPE [5]. In the current study, we noted increased PR length prior to the treatment in all patients. We assume, according to the findings of Spaide et al., that PR elongation may induce PR apoptosis and abnormal PR phagocitosis by the RPE, which is demonstrated in SOCT as hyper-reflective subretinal deposits on the RPE layer [21]. The PR length one month after the laser procedures decreased to 66.2 *μ*m in the MLT group and to 56.8 *μ*m in the CL group, and it can be compared with a typical PR length value of 60 *μ*m in healthy subjects. Twelve months after MLT treatment, PR length remained stable (61.2 *μ*m) and the prevalence of subretinal RPE bulbs declined. Further shortening of PR took place in CL-treated patients, and in almost all of the cases, hyper-reflective deposits persisted. We hypothesize that RPE is not stimulated to excessive PR phagocytosis by micropulsed energy as it happens with continuous-wave energy. Moreover, the PR length stabilization promotes visual function improvement in patients after MLT.

In conclusion, the results showed that MLT was as effective as CL in eyes with chronic CSC in a short time after treatment; however, in long term observation, the micropulse laser led to further improvement of anatomical and functional parameters. In addition, we believe that PR length normalization results in better visual acuity one year after an MLT procedure.

## Figures and Tables

**Table 1:** Descriptive statistics for selected ophthalmological parameters in the studied patients diagnosed as having chronic central serous chorioretinopathy and treated with Micropulse Laser Treatment (MLT) and conventional Continuous-wave Laser (CL).

			Mean ± SD	(Min-max)	Mean ± SD	(Min–max)	Mean ± SD	(Min-max)	*P*
			Baseline		1 month		12 months		
MLT	(*∗*)	Visual acuity	0.53 ± 0.16	6/19–6/7.5	0.84 ± 0.17	6/12–6/6	0.89 ± 0.13	6/9.5–6/6	<0.001
	(†)	Central retinal thickness (*μ*m)	201.5 ± 33.8	132–257	175.7 ± 29.0	108–228	174.1 ± 27.3	108–236	<0.001
	(‡)	Photoreceptors length (*μ*m)	82.8 ± 18.8	56–134	66.2 ± 15.1	40–119	61.2 ± 12.6	40–106	<0.001
	(*∗∗*)	Subretinal fluid (*μ*m)	166.6 ± 96.4	21–430	35.5 ± 27.0	0–127	12.4 ± 27.7	0–126	<0.001

CL	(*∗*)	Visual acuity	0.52 ± 0.20	6/30–6/7.5	0.69 ± 0.18	6/19–6/6	0.71 ± 0.17	6/19–6/6	<0.001
	(†)	Central retinal thickness (*μ*m)	198.8 ± 57.5	64–337	173.9 ± 47.9	65–260	194.9 ± 54.8	65–260	= 0.233
	(‡)	Photoreceptors length (*μ*m)	82.5 ± 23.1	26–123	56.8 ± 18.4	20–90	42.9 ± 8.9	20–55	<0.001
	(*∗∗*)	Subretinal fluid (*μ*m)	199.2 ± 87.9	37–315	27.1 ± 30.3	0–111	12.2 ± 30.1	0–111	<0.001
	Significance level		(*∗*) *P*=0.026		(†) *P*=0.865		(‡) *P*=0.028		(*∗∗*) *P*=0.474

## Data Availability

The data used to support the findings of this study are available from the corresponding author upon request.
